# Synchronous cutaneous malignant peripheral nerve sheath tumor and jejunal gastrointestinal stromal tumor and submucosal angiomyolipoma in type 1 neurofibromatosis: A case report and literature review

**DOI:** 10.1097/MD.0000000000032696

**Published:** 2023-01-20

**Authors:** Kyung Jong Kim, Min Sung Kim, Ran Hong, Sung-Chul Lim

**Affiliations:** a Department of Surgery, College of Medicine, Chosun University, Gwangju, Korea; b Department of Dermatology, College of Medicine, Chosun University, Gwangju, Korea; c Department of Pathology, College of Medicine, Chosun University, Gwangju, Korea.

**Keywords:** angiomyolipoma, gastrointestinal stromal tumor, jejunum, malignant peripheral nerve sheath tumor, skin, type 1 neurofibromatosis

## Abstract

Type 1 neurofibromatosis (NF1) is one of the most prevalent genetic conditions. NF1 is characterized by cutaneous plexiform neurofibromas and café au lait skin pigmentation, and is inherited in an autosomal dominant trait with mutation in the *neurofibromin 1* gene on chromosome 17. Neurofibromin is involved in Ras proto-oncogene regulation. Accordingly, NF1 may lead to malignancies, with a lifetime cancer risk of 60%. Malignant peripheral nerve sheath tumor (MPNST) is the leading cause of mortality due to NF1. The relevance of gastrointestinal stromal tumor (GIST) in NF1 is increasingly being reported in the literature and NF1-associated GIST has been identified to have an alternative molecular pathogenesis.

**Patient concerns:**

A 62-years-old female had a 7 × 5 cm growing back mass in the background of various sized cutaneous neurofibromas with café au lait spots. Computed tomography performed in the workup revealed a 4.1 cm enhancing mass near the ileal mesentery.

**Diagnoses:**

NF1 affected by cutaneous MPNST of the back, and synchronous GIST and submucosal angiomyolipoma (AML) of the jejunum.

**Interventions:**

The patient underwent laparoscopic jejunal mass excision, and excision and flap coverage for the back mass owing to the suspicion of multiple MPNSTs. However, the abdominal masses were diagnosed as GIST and AML following confirmation of the immunohistochemical profiles. Accordingly, the patient was administered adjuvant radiotherapy to the MPNST after surgery.

**Outcomes:**

Symptomatic improvements were achieved, and no subsequent relapses were observed.

**Lessons:**

Although MPNST and GIST are not rare neoplasm in NF1, only 2 case reports have been published on the synchronous occurrence of these tumors. Moreover, no case report has been published on AML in NF1, except 1 renal AML in segmental neurofibromatosis. Identifying the clinical and pathologic significances of the NF1 is important to achieve improved diagnostic accuracy.

## 1. Introduction

Type 1 neurofibromatosis (von Recklinghausen’s disease, NF1) is one of the most prevalent genetic conditions. NF1 is characterized by cutaneous plexiform neurofibromas and café au lait skin pigmentation. Additional diagnostic criteria for NF1 include the presence of optic gliomas, iris hamartomas, and the diagnosis of NF1 in a first-degree relative.^[1]^ NF1 is inherited in an autosomal dominant trait with mutation in the *neurofibromin 1* gene on chromosome 17. Neurofibromin is involved in Ras proto-oncogene regulation. Accordingly, NF1 may lead to the development of malignancies, with a lifetime cancer risk of 60%. Previously, malignant tumors were found to develop 4 times more in NF1 patients than the general population.^[2]^

Malignant peripheral nerve sheath tumor (MPNST) accounts for 5% to 10% of all soft tissue sarcomas. Approximately 50% of MPNSTs occur as NF1, 40% occur sporadically, and the remaining 10% are caused by prior radiation exposure. MPNST is the leading cause of mortality due to NF1.^[3]^

Gastrointestinal stromal tumor (GIST) is the most common mesenchymal tumor in the gastrointestinal tract. However, GIST is a relatively rare disease, occurring in 6 to 15 individuals per million. The molecular pathogenesis of GIST is aberrant tyrosine kinase activity.^[4]^ GIST most commonly occurs in the stomach (50%–70%), followed by the small intestine (25%–36%), colorectum and appendix (5%–7%), and esophagus (1%–3%).^[5]^ Ten to thirty percent of GISTs metastasize, most commonly to the liver (50%–60%) and peritoneum (20%–43%), and in rare cases, to the bones and lung (10%) outside the peritoneum.^[6]^ GIST is a relatively common disease in patients with NF1, and approximately 90% of GIST associated with NF1 occurs in the small intestine. However, sporadic GIST most commonly occurs in the stomach.^[7]^ GIST associated with NF1 has an alternative molecular pathogenesis.^[8,9]^ Unlike sporadic GIST, GIST associated with NF1 appears to respond less well to tyrosine kinase inhibitors due to a lack of mutation in the *c-kit gene* or *PDGFRA gene*.^[10]^

Although MPNST and GIST are not rare neoplasm in NF1, only 2 case reports on the synchronous occurrence of these tumors have been published.^[11,12]^

Angiomyolipoma (AML) is a benign mesenchymal hamartoma that occurs primarily in the kidney. Extra-renal AML is most common in the liver but also occurs in the gastrointestinal tract. Notably, extra-renal AML has been reported to occur in the stomach, duodenum, small intestine, and colon. All cases of AML in the small intestine were found in the ileum, with no reported case of AML in the jejunum.^[13]^ There is no known association between NF1 and AML; however, 1 case of segmental neurofibromatosis with renal AML has been reported.^[14]^

Here, we sought to report a case of NF1 affected by cutaneous MPNST of the back and synchronous GIST and submucosal AML of the jejunum. Owing to its rarity and unusual clinical presentation, this is the first case report of synchronous MPNST, GIST, and extrarenal AML in NF1. Herein, a review of the relevant literature is also presented.

## 2. Case presentation

**Figure 1.**
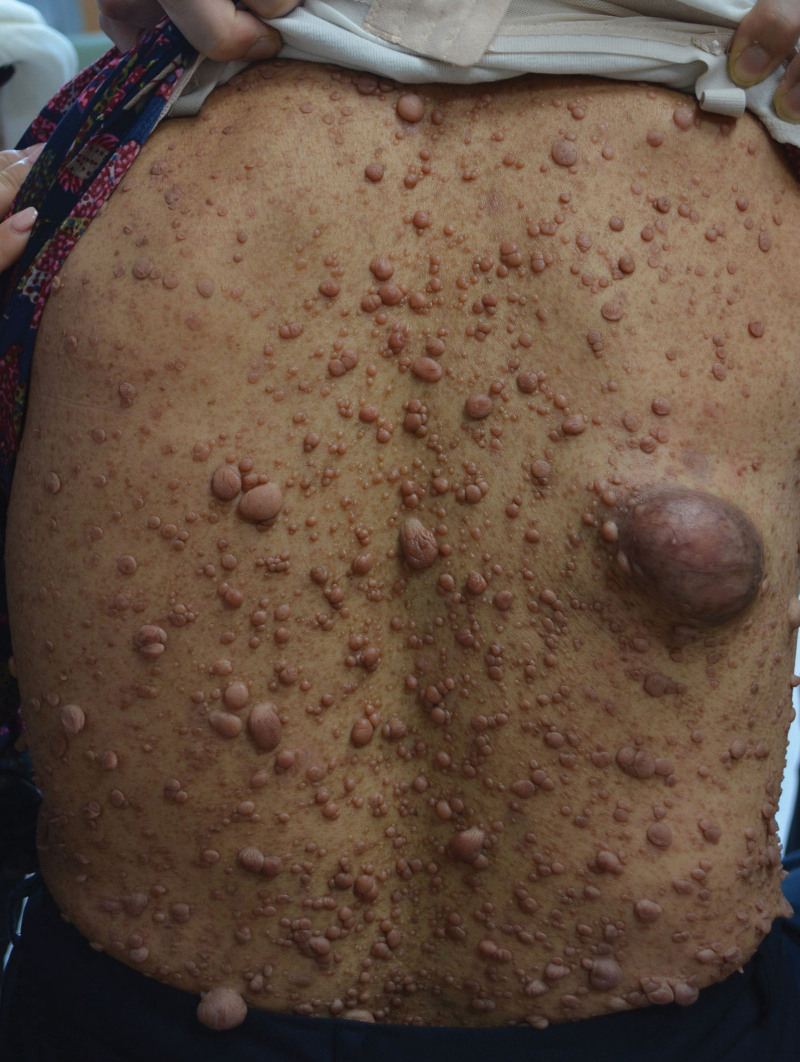
Image of neurofibromas on the body surface.

A 62-years-old female was admitted to the hospital with the chief complaint of a mass on the right side of the back. The patient had long-standing neurofibromas on her body. Further, numerous café au lait skin pigmentations were observed in the background of these tumors. After the patient revealed the neurofibromas on her skin to the medical staff, she was diagnosed with neurofibromatosis. The mass on the patient’s back was 7 × 5 cm in size at the time of measurement. The mass was identified to be continuously growing and was accompanied by intermittent pain that was becoming increasingly worse (pain scale: 4/10; Fig. 1). Only the youngest daughter of the patient was diagnosed with neurofibromatosis. No other family history of this disease was reported.

Based on the above findings, the patient was presumed to develop this malignant transformation from a neurofibroma that occurred long ago.

**Figure 2.**
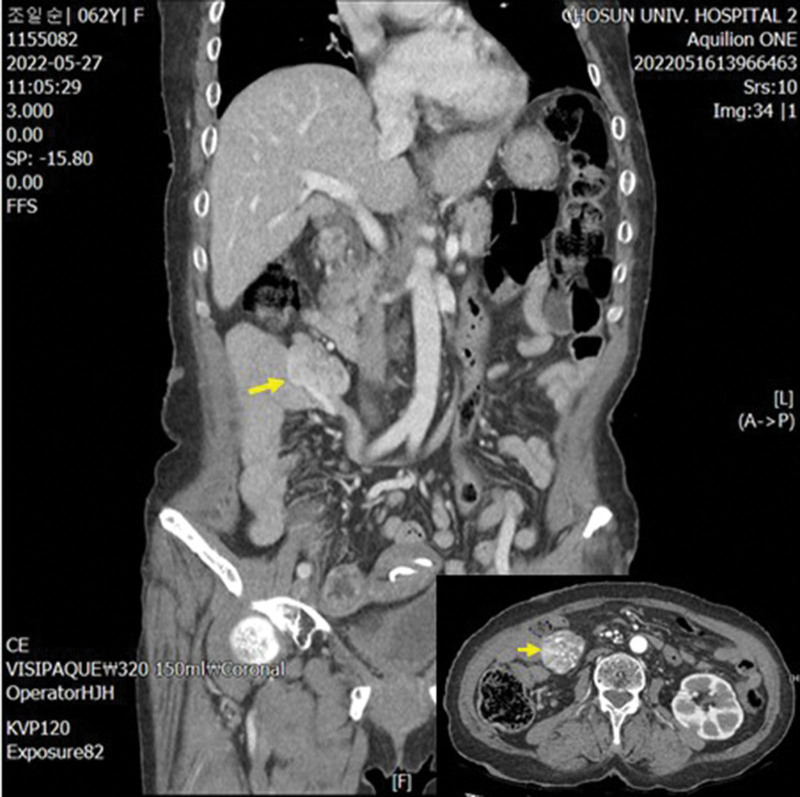
Abdominal and pelvic CT findings. An enhancing mass of approximately 4.1 cm (arrows) was identified near the ileal mesentery. CT = computed tomography.

A computed tomography of all organs, including the brain, was performed to check for abnormalities of other organs prior to surgical treatment of the mass on the back. The result revealed a 4.1 cm-long enhancing mass near the ileal mesentery, which was radiologically suspected as a neurogenic or neuroendocrine tumor (Fig. 2). Cysts were observed in both kidneys. Diffuse cortical atrophy and moderate ventricle enlargement were identified via the brain scan; however, there were no abnormal findings in the brain parenchyma or in other organs (Fig. 2).

No hematologic abnormalities were identified in the preoperative check.

**Figure 3.**
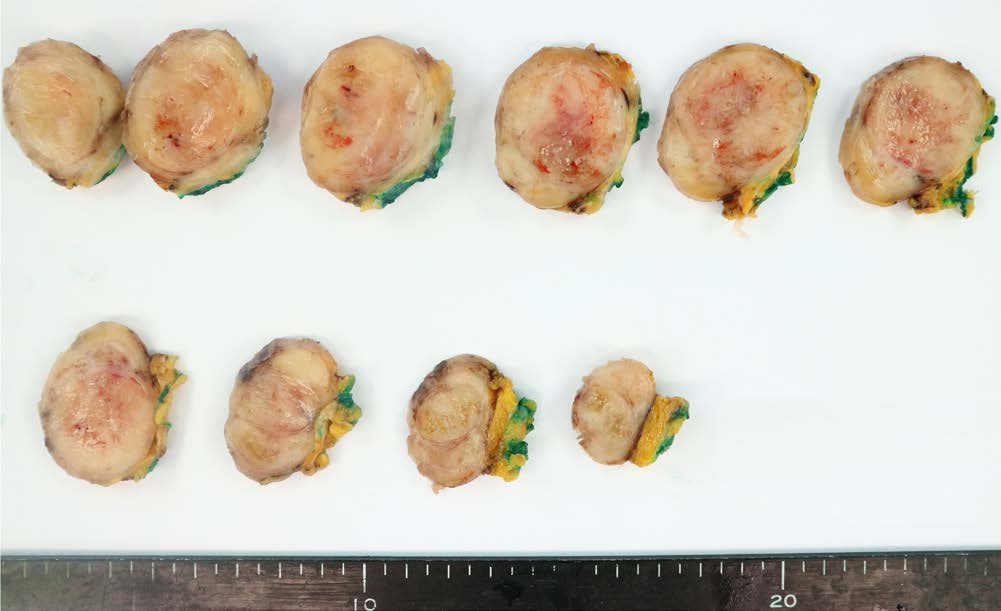
Gross findings of the back mass.

**Figure 4.**
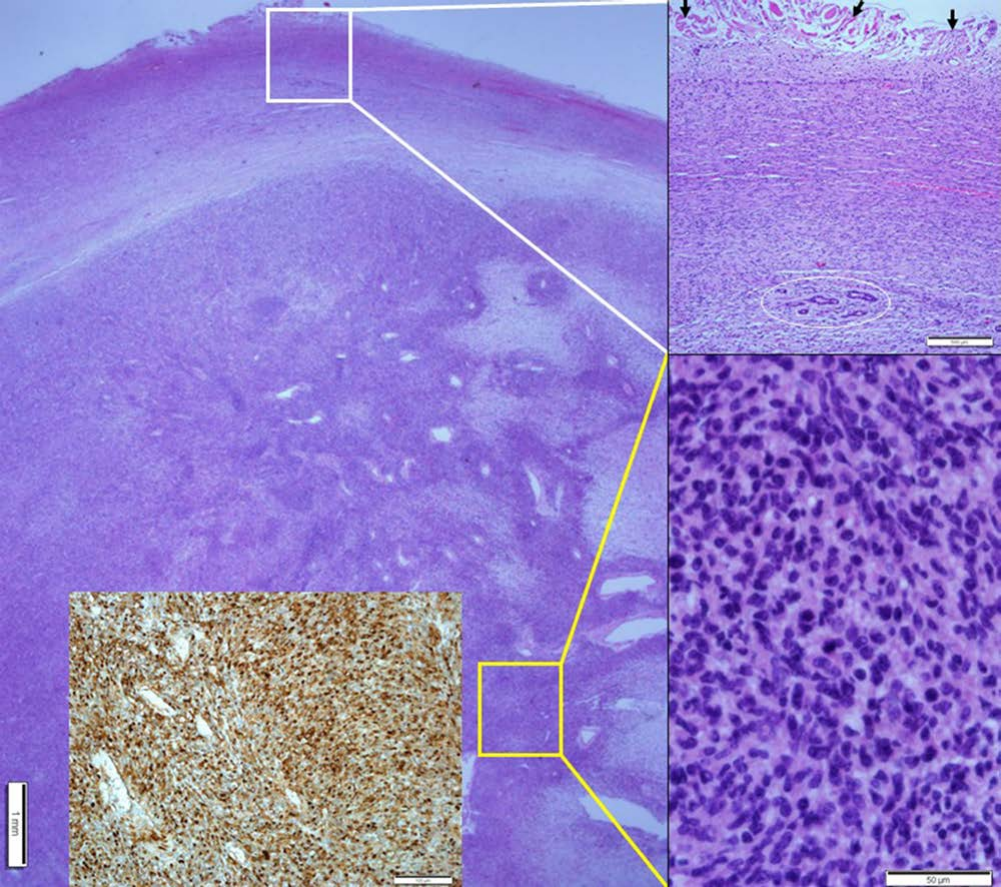
Histopathologic findings of cutaneous MPNST. A well-circumscribed nodular mass showing dense cellular fascicles alternate with myxoid regions from the dermis to the deep subcutaneous layer. At a higher magnification, the section in the white box was found to have less cellular spindle cells with no cellular atypia. Entrapped skin appendages were identified in the circle. Arrows indicate dermal collagen at the resection margin. At a higher magnification, the section in the yellow box was found to have more cellular spindle cells with cellular atypia and occasional atypical mitoses. Inset: Positive immunohistochemical staining for the *S*-100 protein. MPNST = malignant peripheral nerve sheath tumor.

Mass excision and flap coverage were first performed on the patient’s back mass. The excised mass was well circumscribed and was 4.0 × 3.5 cm in size. No cross-sectional necrosis was identified; however, a bleeding site was recognized (Fig. 3). Through microscopic evaluation, the mass was identified as a well-circumscribed nodular mass dispersed from the dermis to the deep subcutaneous layer with dense cellular fascicles alternating with myxoid regions. No necrosis was observed. The tissues constituting the marginal zone of the mass were composed of less cellular spindle cells, and no cellular atypia was found. However, the tissues constituting the inner (core) part had higher cellular spindle cells, which increased the nuclear to cytoplasmic ratios. Further, hyperchromasia was present. Accordingly, the cellular atypia is considered to be accompanied by slightly increased numbers of mitotic figures (<3/10 high power fields [HPFs]). Tumor necrosis was absent, and occasional atypical mitoses were observed. Most tumors contained interlacing bundles of collagen and a moderate amount of extracellular myxoid material. Most tumor cells were long and spindle-shaped and had slender, bipolar processes and wavy, dark-staining nuclei. A few large and pleomorphic tumor cells were observed. The margin was involved by tumor cells. Immunohistochemical staining of the tumor cells revealed positive signals for the *S*-100 protein and negative signals for *α*-smooth muscle actin, CD34, and c-Kit (CD117). Based on these findings, the back mass was diagnosed as MPNST, low grade (histologic grading by Fédération Nationale des Centres de Lutte Contre le Cancer: FNCLCC grading system,^[15]^ grade 1; Fig. 4). The clinical stage was stage I (*T*_1_ N_0_ *M*_0_).

The patient received adjuvant radiotherapy for the MPNST at 4 weeks after the operation as tumor cells were involved in the resection margin of the patient’s mass. The radiation dose was 180 to 200 cGy daily in 5 fractions/weeks, resulting in a total dose of 45 to 50 Gy/5 weeks.

Laparoscopic mesenteric mass removal was then performed for the mesenteric mass. Two masses on the jejunum were identified during the operation, and segmental resection of the jejunum was performed for each.

**Figure 5.**
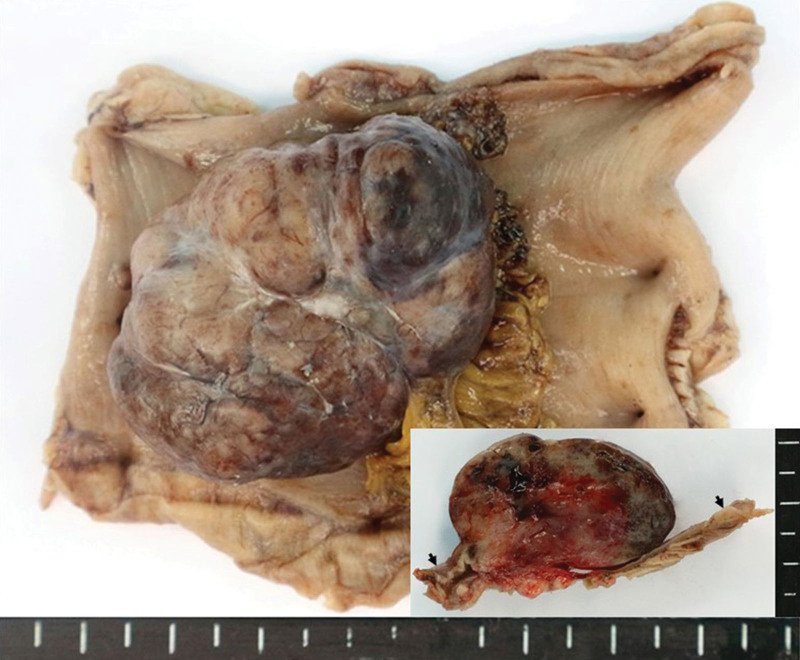
Gross findings of the jejunal GIST. Arrows indicate serosal surface. GIST = gastrointestinal stromal tumor.

**Figure 6.**
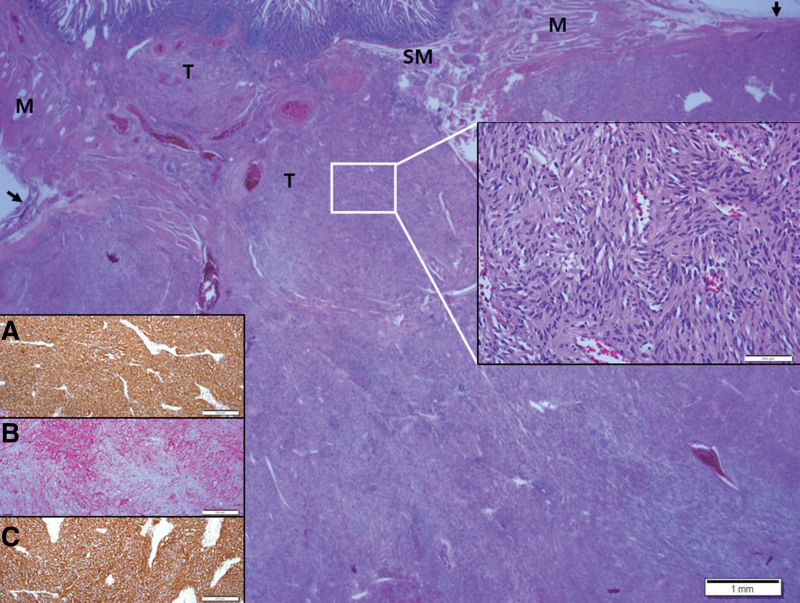
Histopathologic findings of GIST in the jejunum. A well-circumscribed protruding tumor (T) from the submucosa (SM) to serosa (arrows) was identified. M: muscle proper. At a higher magnification, the section in the white box was found to have spindle cells with cigar-shaped nuclei and pale eosinophilic cytoplasm with indistinct membrane. No atypia and mitosis were noted. Positive immunohistochemical staining for C-Kit (A), CD34 (B), and Dog 1 (C). GIST = gastrointestinal stromal tumor.

The mass of 4.5 × 4.0 × 4.0 cm protruding toward the serosa was well-circumscribed and appeared dark red due to bleeding (Fig. 5). Some of the lesions were referred for frozen diagnosis during the operation. The lesions were diagnosed as malignant tumors as high cellularity and occasional atypical mitoses were identified. The possibility that the lesions may be the same as the MPNST on the patient’s back was reported. The biopsy results of the samples sent after the operation revealed that the mass was located in the submucosa, muscle, and serosa of the jejunum and consisted of spindle cells with cigar-shaped nuclei and pale eosinophilic cytoplasm with indistinct membranes. No atypia and tumor necrosis were noted. Mitoses were very rarely observed with < 2/50 HPFs. The margin was free of tumor cells. Positive immunohistochemical staining was observed for c-Kit, CD34, and Dog 1 while negative staining was observed for *α*-smooth muscle actin, *S*-100 protein, and CD31. Based on these observations, the mass protruding toward the serosa was diagnosed as GIST, low risk (pT2, prognostic category 2) category (Miettinen and Lasota’s algorithm^[16]^; Fig. 6).

No alteration was identified in the *c-kit gene* (exons 8, 9, 11, 13, and 17) and *PDGFRA gene* (exons 12, 14, and 18) mutation studies of GIST and the surrounding normal mucosa. Therefore, this case is not a familial GIST case resulting in a germline mutation in the *c-kit* or *PDGFRA gene*.

The patient was followed-up without tyrosine kinase inhibitor administration as the tumor case is low-risk GIST with wild-type *c-kit* and *PEGFRA genes*.

**Figure 7.**
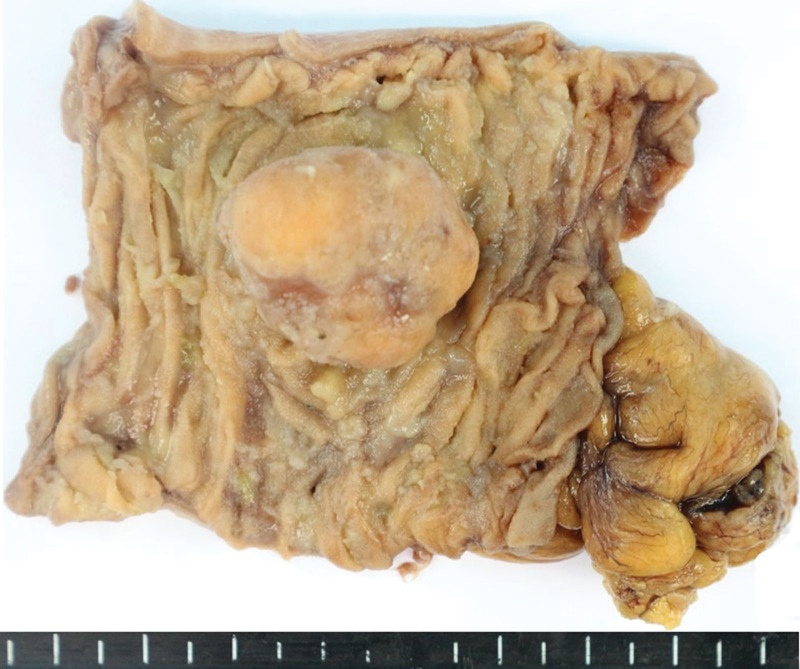
Gross findings of the jejunal submucosal AML. AML = angiomyolipoma.

**Figure 8.**
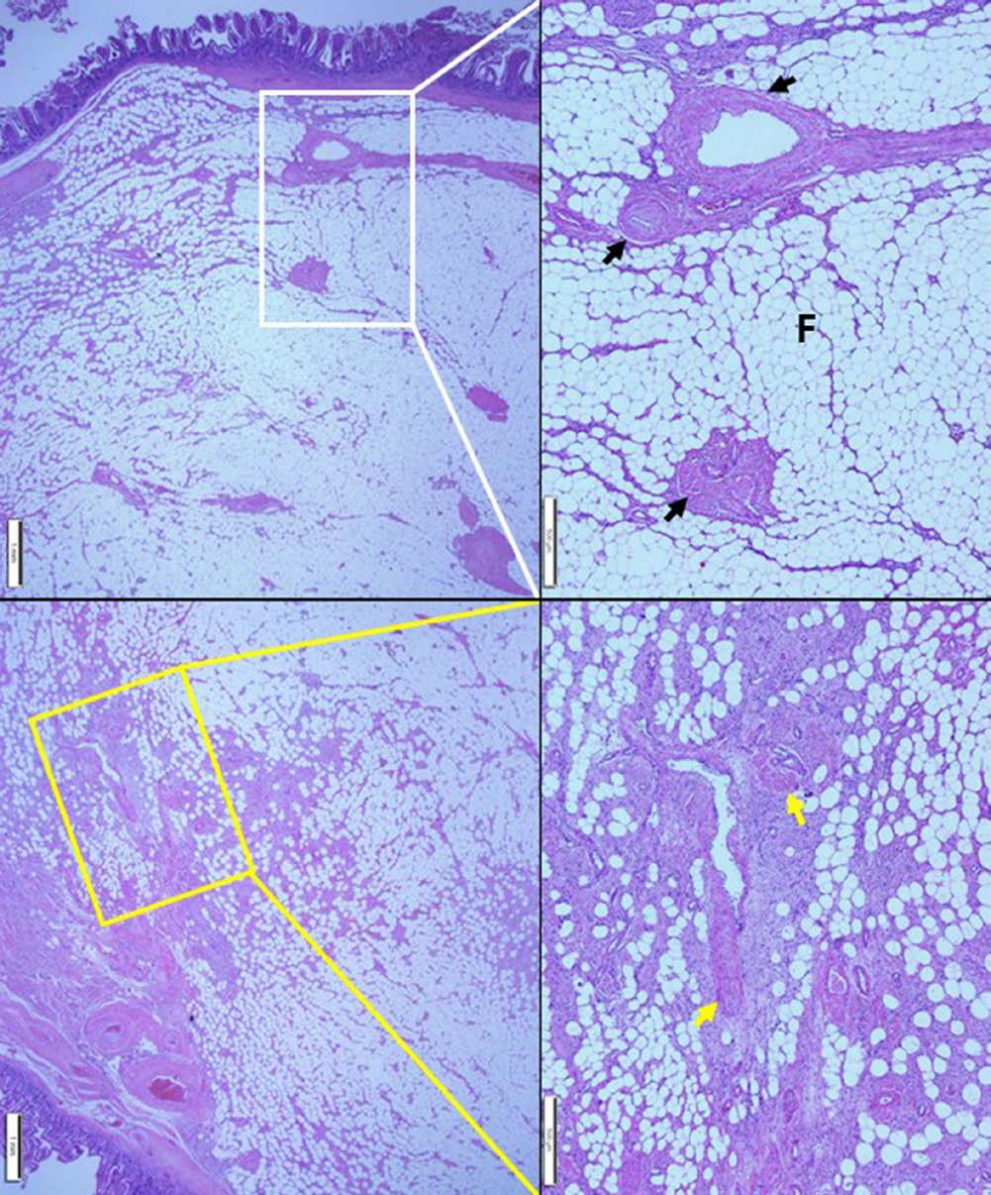
Histopathological findings of jejunal AML. A well-circumscribed luminal polypoid tumor involving the submucosa was identified. At a higher magnification, the section in the white box was found to have variable-sized thin and thick-walled blood vessels (black arrows) and mature adipocytes (F). Higher magnification of the section in the yellow box revealed several blood vessels, chunky smooth muscle bundles (yellow arrows), and mature adipocytes. AML = angiomyolipoma.

**Figure 9.**
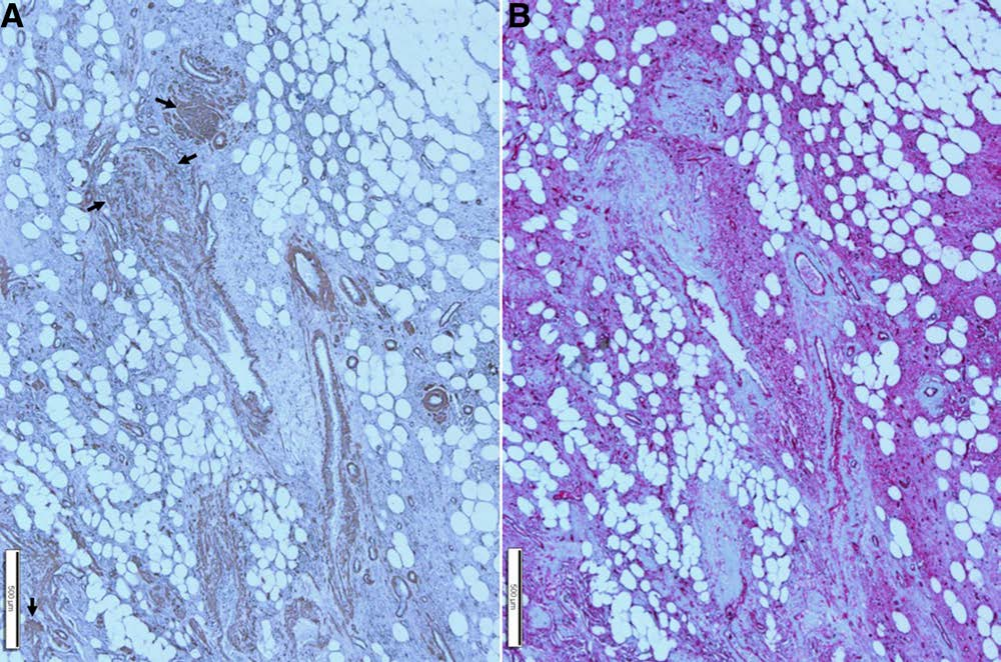
Immunohistochemical findings of the area outlined by a yellow box in Fig. 8. (A): Immunohistochemical staining for smooth muscle actin revealed thin and thick-walled blood vessels and smooth muscle bundles (arrows). (B): Immunohistochemical staining for CD34 revealed thin and thick-walled blood vessels and intervening spindle cells.

The remaining mass of the jejunum was a well-circumscribed 2.5 × 2.5 × 2.2 cm polypoid mass protruding toward the lumen. The cross-section of the mass was yellow, with no bleeding or necrosis found. The macroscopic mass was thought to be a submucosal lipoma (Fig. 7). According to the microscopic observation, the mass was identified to be located in the submucosa and consisted of variable-sized thin and thick-walled blood vessels and mature adipocytes. Higher magnification revealed several blood vessels, chunky smooth muscle bundles, and mature adipocytes. No atypia and mitosis were noted. The margin was also free of tumor cells (Fig. 8). The immunohistochemical staining revealed positive *α*-smooth muscle actin in thin and thick-walled blood vessels and smooth muscle bundles. The endothelial lining of the thin and thick-walled blood vessels and surrounding spindle cells were CD34 positive (Fig. 9). Accordingly, the luminal polypoid mass of the jejunum was diagnosed as AML.

No signs of recurrence have been observed after 11 months of surgery with computed tomography follow-up.

This study was approved by the institutional review board of the Chosun University Hospital (Permission number: CHOSUN 2022-08-003). Written informed consent was obtained from the patients for the publication of this case report and the accompanying images.

## 3. Discussion

NF1 is one of the most common autosomal dominant genetic disorders, with an occurrence rate of 1 in 2500 to 3000 individuals.^[1]^ Mutation in the *NF-1 gene* causes abnormalities in the tumor suppressor genes, which increases the incidence of malignancies and causes various systemic manifestations. Neurofibromas, carcinoid tumors, and GISTs are more common than in the general population.^[17]^ Approximately half of MPNST cases are associated with the *NF-1 gene*, which poses a higher risk of local recurrence and distant metastasis. However, MPNST in the gastrointestinal tract is uncommon,^[3,18]^ and only 1 case of gastric MPNST in NF1 has been reported.^[19]^

The present case is considered an MPNST resulting from malignant transformation in one of the numerous neurofibromas in a patient with long-standing NF1.

GISTs are the most common mesenchymal tumors in the gastrointestinal tract. GISTs most commonly occur in the stomach and are relatively rare in the small bowel. However, approximately 90% of the GISTs associated with NF1 occur in the small intestine and only 5.4% occur in the stomach.^[7]^ In the current case, an operation was performed after the diagnosis of a back mass as MPNST. A spindle cell tumor suspected of cellular atypia in the intraoperative frozen diagnosis was similar to the lesion on the back. Further, MPNST was proposed to have occurred in the jejunum.

GIST is microscopically categorized into 3 histologic subtypes: pure spindle cell type (70%), pure epithelioid type (20%), or mixed morphology type (10%).^[6]^

According to the reported data, 27.6% of GIST was identified as a low-risk category (Miettinen and Lasota’s algorithm); however, most metastases occurred within 2 years (27.7%) if the tumor size was larger than 10 cm of the non-spindle cell histologic type, mitotic count over 5/5 mm^2^, myxoid change, and the presence of mucosal invasion. Relapse (8.1%) occurred within 7 years and was associated with myxoid change.^[20]^

The risk of metastasis or recurrence was considered very low in the current case of pure spindle cell type with the absence of myxoid change and mucosal invasion.

Synchronous development of multicentric MPNST is unusual, with an incidence rate of 1.4% in NF1 and 0.5% in the entire cohort of MPNST. Further, no asynchronous multicentric MPNST has been reported.^[21]^

The routine histopathologic examination after the operation revealed that the jejunal serosa mass consisted of spindle cells with cigar-shaped nuclei and pale eosinophilic cytoplasm with indistinct membrane, displaying some differences from MPNST. Immunohistochemical staining revealed positive Dog 1, which could represent GIST. However, a previous report revealed that MPNST could also be positive.^[22]^ Thus, S-100 protein staining was performed; however, a negative result was obtained. As benign neurogenic tumors are generally positive for the *S*-100 protein but MPNSTs may be negative for the *S*-100 protein, CD34 and C-Kit staining were added to confirm a strong positive result.

Although MPNST is uncommon in the gastrointestinal tract, the diagnosis was confirmed as it was consistent with GIST based on immunohistochemical staining.

There have been reports of GISTs associated with NF1 occurring as a different molecular pathogenesis of GISTs in the general population,^[4,23]^ such reports have important therapeutic implications. GISTs associated with NF1 have either a *c-kit* or *PDGFRA gene* identified as wild-type. Further, as most cases of GISTs associated with NF1 appear as the low-risk type, tyrosine kinase inhibitors are not administered to the general population presenting GIST.^[4,8]^

GISTs associated with NF1 have no *c-kit* or *PDGFRA gene* mutations; however, c-Kit and PDGFRA expression are observed via immunohistochemical staining, suggesting that GIST can develop due to increased signal transduction through the MAP-kinase pathway owing to the somatic mutation of wild-type NF1 allele in tumors, without *c-kit* or *PDGFRA gene* mutations.^[4,8,23,24]^ No *c-kit* or *PDGFRA gene* mutations were found; however, strong expression of c-Kit was observed in the present case based on immunohistochemical staining.

Small bowel tumors are rare, and jejunal and ileal tumors are mainly malignancies, such as carcinoma, lymphoma, and GIST.^[25]^

AML is the first rare tumor reported in the kidney. AML occurs in 0.3% of the population and is often associated with tuberous sclerosis. Extra-renal AML is very rare and most commonly occurs in the liver, with an even rarer occurrence in the gastrointestinal tract. Only few reports of the occurrence of extra-renal AML in the colon, stomach, and small bowel have been published.^[13,26–29]^ However, no reports of NF1 being accompanied by AML have been published, with only 1 case of renal AML reported to occur in segmental neurofibromatosis.^[14]^

AML is a tumor composed of varying proportions of mature adipocytes, smooth muscle, and irregular blood vessels. AML has been observed to occur in the duodenum and ileum but not in the jejunum, as revealed in the current case. The association between NF1 and AML cannot be explained due to the lack of cases of AML in NF1. However, whether NF1 is related to AML should be determined based on the nature of NF1 and its association with oncogenesis in various organs.

## 4. Conclusions

NF1 causes multiple types of tumors; however, to the best of our knowledge, only 2 cases of synchronous occurrence of MPNST and GIST have been reported in the English literature. AML, which is very rare in the gastrointestinal tract and has never been reported to occur in the jejunum, was found to occurred with NF1, in addition to synchronous MPNST and GIST. Accordingly, this is the first report of the simultaneous occurrence of MPNST, GIST, and AML in NF1. The relationship between NF1 and AML can be determined if more cases are analyzed in the future. A broad clinical and pathological understanding of NF1 will not only improve diagnostic accuracy but will be important for the selection of suitable therapeutic agents.

## Acknowledgements

The present study was supported by grants from the Chosun University (2022).

## Author contributions

**Data Curation:** Kyung Jong Kim, Min Sung Kim.

**Funding acquisition:** Sung-Chul Lim.

**Methodology:** Sung-Chul Lim, Ran Hong.

**Project administration:** Sung-Chul Lim.

**Supervision:** Sung-Chul Lim.

**Validation:** Sung-Chul Lim, Kyung Jong Kim, Min Sung Kim.

**Writing – original draft:** Sung-Chul Lim.

**Writing – review & editing:** Ran Hong, Sung-Chul Lim.
